# A feature selection approach for identification of signature genes from SAGE data

**DOI:** 10.1186/1471-2105-8-169

**Published:** 2007-05-22

**Authors:** Junior Barrera, Roberto M Cesar, Carlos Humes, David C Martins, Diogo FC Patrão, Paulo JS Silva, Helena Brentani

**Affiliations:** 1 Instituto de Matemática e Estatística, Universidade de São Paulo, Rua do Matão 1010, São Paulo, Brazil; 2 Hospital do Cancer A. C. Camargo, Rua Prof. Antonio Prudente 211, São Paulo, Brazil

## Abstract

**Background:**

One goal of gene expression profiling is to identify signature genes that robustly distinguish different types or grades of tumors. Several tumor classifiers based on expression profiling have been proposed using microarray technique. Due to important differences in the probabilistic models of microarray and SAGE technologies, it is important to develop suitable techniques to select specific genes from SAGE measurements.

**Results:**

A new framework to select specific genes that distinguish different biological states based on the analysis of SAGE data is proposed. The new framework applies the bolstered error for the identification of strong genes that separate the biological states in a feature space defined by the gene expression of a training set. Credibility intervals defined from a probabilistic model of SAGE measurements are used to identify the genes that distinguish the different states with more reliability among all gene groups selected by the strong genes method. A score taking into account the credibility and the bolstered error values in order to rank the groups of considered genes is proposed. Results obtained using SAGE data from gliomas are presented, thus corroborating the introduced methodology.

**Conclusion:**

The model representing counting data, such as SAGE, provides additional statistical information that allows a more robust analysis. The additional statistical information provided by the probabilistic model is incorporated in the methodology described in the paper. The introduced method is suitable to identify signature genes that lead to a good separation of the biological states using SAGE and may be adapted for other counting methods such as Massive Parallel Signature Sequencing (MPSS) or the recent Sequencing-By-Synthesis (SBS) technique. Some of such genes identified by the proposed method may be useful to generate classifiers.

## Background

Using high-throughput molecular approaches, mainly microarrays, several groups of genes have been identified to be associated with cancer [1-8]. Molecular profiles have been associated with specific histologic and prognostic tumor subgroups, but the number of genes in the different profiles is too high to be used as signatures for classification. The limited amount of available human tissues and the cost of gene expression screening projects yield the search for classifiers that only depend on small sets of genes. In the pattern recognition literature, the problem of finding a subspace of variables that is enough to distinguish classes of patterns is known as dimensionality reduction. Despite the existence of a large literature about dimensionality reduction [9], most of it does not apply for classification from gene expression vectors due to lack of observed data. Typically, we have spaces of some thousands of genes and would like to get subspaces of two or three genes from the observation of some dozens of expression vectors. To overcome this difficulty, the strong genes technique adopts a probabilistic model for the random vector distribution of each class: the union of round uniform spread functions, which are estimated from the observed data [10]. The strong genes technique was proposed in [10] and used for glioma classification with microarrays. The estimated model is projected onto a sub-space of a small number of variables *n *where the error of the optimum linear classifier is computed. This procedure is repeated for all subspaces of *n *variables and the genes *n*-tuple quality is evaluated by the corresponding classification error: the best *n*-tuples generate the best separators and, therefore, those that have smallest errors. The original version of this procedure requires hours in a supercomputer. Nevertheless, an approximation technique was recently developed which requires just some minutes in a conventional desktop computer [11].

A large scale approach widely used in gene expression studies is the Serial Analysis of Gene Expression (SAGE) [12]. SAGE uses a very different approach compared to microarrays for measuring mRNA levels. First, double stranded cDNA is created from the mRNA. A single 10 base pair "sequence tag" is cut from a specific location in each cDNA. The sequence tags are concatenated into a long double stranded DNA which can then be amplified and sequenced. The expression of a gene in a given experiment is estimated just by counting the number of tags in the sequence corresponding to that gene, thus providing absolute transcript numbers and allowing statistical comparisons of data from multiple laboratories.

Vêncio *et al*. [13] modeled SAGE gene expression measurements by a Beta distribution and applied Bayesian estimation to calculate the corresponding credibility interval, thus providing an important tool for statistical analysis of SAGE data. It is important to note that, in contrast to microarray where the data formation probabilistic model is unknown, the model representing SAGE gene expression provides further statistical information that allows a more robust analysis. The additional statistical information provided by the probabilistic model is incorporated in the methodology described in the paper. We explored the SAGE statistical model and modified the strong genes technique in order to make it suitable for distinguishing classes of patterns from SAGE measurements. Due to important differences in the probabilistic models of microarray and SAGE technologies, we propose the concept of subspace credibility. This concept, based on the aforementioned credibility intervals of SAGE measurements introduced in [13], is used to complement the application of the strong genes technique. The credibility gives a measure of the distance between the two classes according to the credibility interval model. The best chosen subspaces are those that have both minimum bolstered error (computed by the strong gene technique) and maximum credibility. A score taking into account these two measures is proposed in the present work.

Therefore, the resulting new methodology allows the application of the strong genes technique to SAGE data in order to select gene subspaces in a consistent way under the perspective of the aforementioned SAGE gene expression measurements model. The double criterion allows gene selection in a more feasible way when SAGE data is involved. The proposed technique was applied to distinguish glioma tumors and the results are reported. According to the World Health Organization (WHO) classification of brain tumors, gliomas are divided in low-grade (grades I and II) and high-grade (grades III and IV) tumors. Low-grade tumors are well-differentiated, slow-growing lesions. Grade I tumors are well-circumscribed and often curable, whereas grade II, III and IV tumors are diffuse, infiltrating lesions. Grade II tumors have a marked potential overtime for progression towards a high-grade malignant tumor.

## Results

The introduced methodology was applied to SAGE data to identify genes that were putatively related to neurological tumor progression. Table 1 shows the different anatomopathological comparisons and the number of libraries in each class.

**Table 1:** Anatomopathological comparisons. Anatomopathological comparisons and the number of libraries in each class (*n *= normal, *a *= astrocytoma grades II and III, *g *= glioblastoma, *a*2 = astrocytoma grade II, *a*3 = astrocytoma grade III). The symbol "O" represents samples of the first group, and "X" represents samples of the second group in Figures 2 and 3.

Comparison label	Normal (2)	Astrocytoma II (4)	Astrocytoma III (9)	Glioblastoma(9)
1 (normal × glio)	O	-	-	X
2 (normal × astro, glio)	O	X	X	X
3 (astro 2 × astro 3)	-	O	X	-
4 (astro 2 × astro 3, glio)	-	O	X	X
5 (astro 3 × glio)	-	-	O	X
6 (astro × glio)	-	O	O	X

**Figure 1:**
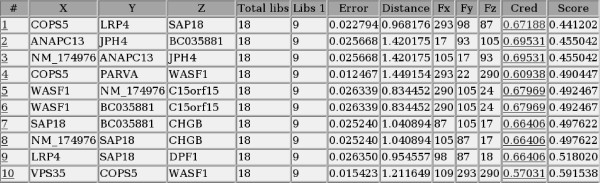
**Sample output table in HTML format**. Sample of the output table produced by the system in HTML format (*astrocytoma III *× *glioblastoma *experiment).

The first 1000 ranked triples according to the introduced approach (refer to the Methods section below) were considered out of about 908 billion possible triples. The sets of triples obtained in all comparisons ordered by the bolstered error and by the introduced score (Equation 3, see Methods section) are available at the supplemental material web-site [14]. Figure 1 shows a list of the first 10 ranked triples in the normal × glioblastoma comparison. When the compared classes are very distant with respect to the error, it is very difficult to choose the best triple from the first 1000 ones because either the errors are very small (zero for six or more significant digits) or they are very similar. This fact is due to the small sample size. When the distance between classes is smaller, although the triples may be ranked using the error, it is still difficult to choose the best triple since the error variation between the triples is too small. In order to circumvent these problems, it is important to take into account the credibility measure for each expression measurement based on the total number of tags counted in each library. This is accomplished by the score measure defined by Equation 3 which incorporates the error and the credibility measures to rank the triples. The application of the score shows that, despite of a low error and a large distance, some triples are no longer ranked in the top positions as they were by only using the error criterion because the credibility value is too low. This means that the proximity of some samples of one group with samples of the other may be large considering the location, volume and edge size of each credibility interval box. Figure 3 shows an example of a three-dimensional plot representing the credibility interval boxes for the best triple produced by the system for the *astrocytoma III *vs *glioblastoma *experiment. Note that this triple reliably separates the two classes taking into account the credibility boxes.

**Figure 2:**
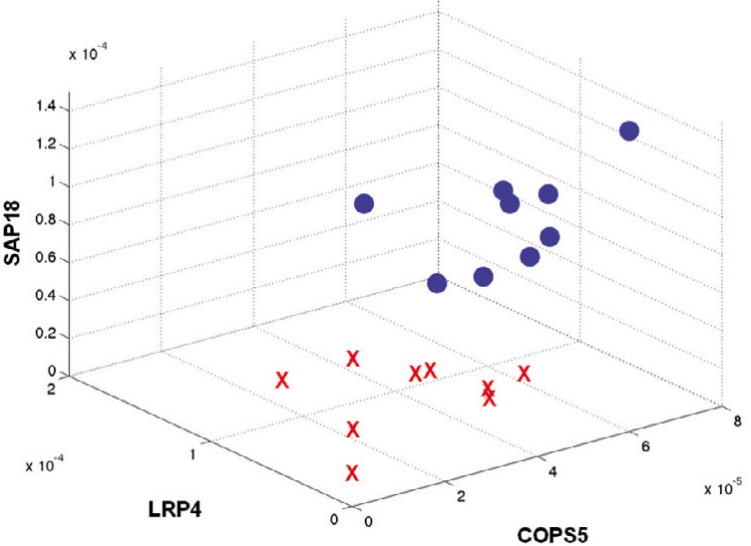
**Plot for the best triple of the astrocytoma III × glioblastoma experiment**.Three-dimensional plots= for the best gene triple produced by the system (astrocytoma III × glioblastoma experiment) without credibility interval boxes.

**Figure 3:**
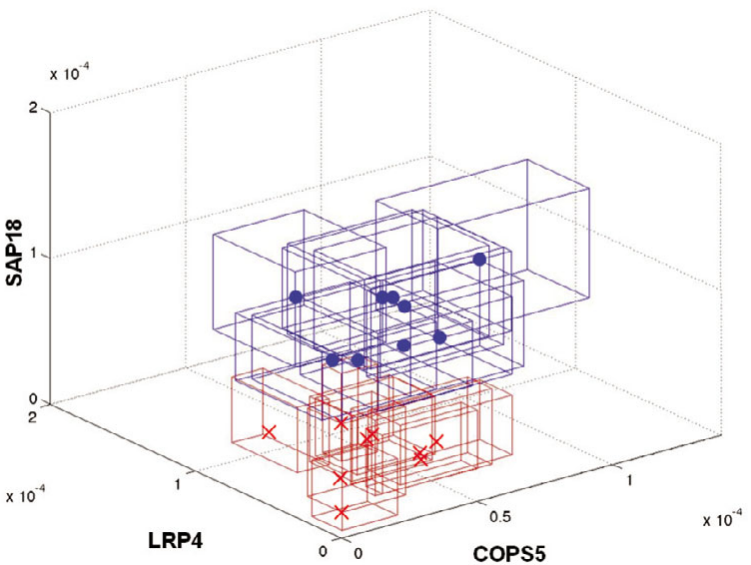
**Plot for the best triple of the astrocytoma III × glioblastoma experiment**. Three-dimensional plots= for the best gene triple produced by the system (astrocytoma III × glioblastoma experiment) with credibility interval boxes.

Table 2 presents the bolstered error along with its standard deviation and the average distance of the nearest point from the hyperplane, for the first 50 triples from each comparison, ranked by the bolstered error and by the score, as well as a list of the ten most frequent genes along the 50 best triples. The first comparison was normal brain against glioblastoma multiform (GB) since we expected the largest difference in this combination of classes. According to Table 1, this comparison comprises 2 versus 9 libraries for normal and GB, respectively. As expected for the first comparison, the obtained bolstered error is the smallest among all class comparisons and the distance is the largest one. In the same table, the second ranked comparison is number two (normal × all tumors), as expected. The error and average distance of triples that separate normal brain from neoplasic tissue are almost ten times larger than the average distance between tumors. As far as the tumor classes are concerned, the distance between grades II and III is the highest. It may be observed from comparisons 3, 4, 5 and 6 in Table 2 that the average distances tend to be smaller, reflecting the glioblastoma progression from *astrocytoma *grade II to III. *Astrocytoma *grade III seems to be between *astrocytoma *grade II and *glioblastoma*, as well as it seems to be closer to *glioblastoma *than to *astrocytoma *grade II, although the differences are very small.

**Table 2:** Summary of comparisons. For each comparison, this table shows the 10 most frequent genes along the first 50 best triples ranked by bolstered error and score. Avg. Error = bolstered error; Avg. Dist. = average distance of the nearest point from the hyperplane; std. dev. = standard deviation.

Comparison	normal × glio	normal × astro, glio	astro 2 × astro 3	astro 2 × astro3, glio	astro 3 × glio	astro 2, astro3 × glio
**Ranked by avg. bolstered error**						

Avg. Error (std. dev.)	0.0 (0.0)	0.0 (0.0)	0.003 (0.0)	0.016 (0.003)	0.022 (0.004)	0.031 (0.003)
Avg. Dist. (std. dev.)	59.217 (90.175)	21.76 (0.35)	2.194 (0.297)	1.095 (0.222)	1.115 (0.256)	0.785 (0.199)
1	ITPKA	CPNE7	SCN4B	LOC646999	COPS5	COPS5
2	CPNE7	AK055475	GPX2	RPM2B	WASF1	ZDHHC22
3	NDFIP2	TAIP-2	ZNF233	AK090819	SAP18	BC035881
4	KIAA1345	PCDH9	APBA3	RALGPS1	VPS35	WASF1
5	HLF	LCE2D	HSU79275	RP1-32F7.2	CHGB	PRPF39
6	FLJ31636	AK095013	LRRC50	HSU79275	FLJ31818	LHFPL2
7	BX648951	C1QL2	HNF4G	LRRC50	MORF4L1	ZNF644
8	CYP7B1	LOC36003	RAET1E	FLJ20323	FLJ39538	POLDIP3
9	BC042456	GPR97	AX090819	DUSP9	ZNF233	ZBTB5
10	PIK3C3	GSTO2	MRC2	WDR35	DNASE2	LRP12

**Ranked by score**						

Avg. Error (std. dev.)	0.0 (0.0)	0.0 (0.0)	0.006 (0.003)	0.021 (0.006)	0.028 (0.007)	0.033 (0.005)
Avg. Dist (std. dev.)	18.48 (1.17)	10.03 (0.23)	1.91 (0.23)	0.91 (0.32)	1.02 (0.28)	0.83 (0.21)
1	RPH3A	CALM3	APBA3	RRM2B	WASF1	COPS5
2	ITPKA	SEPT5	PARVA	RALGPS1	SAP18	ZDHHC22
3	HLF	EEF1A2	BC023565	PDE8A	COPS5	BC035881
4	NDFIP2	LCE2D	BC015762	BDP1	ZDHHC22	SAP18
5	SH3GL2	PPP2R4	DNASE2	RP1-32F7.2	BC035881	ANAPC13
6	SYT13	SULT4A1	KBTBD6	TMEFF1	LRP4	LRP4
7	BCL2L2	ATP1A3	KCTD9	BC023565	JPH4	JPH4
8	MGC34830	VMP	ZNF354C	BC015762	CHGB	PRPF39
9	PLEKHB2	PNMA6A	C15orf29	ZNF354C	ANAPC13	LHFPL2
10	DSCR1L1	FBXO2	SHOC2	C15orf29	POLDIP3	SSR3

According to Table 2, the credibility provides robustness to the selection since the standard deviation of the distances and of the bolstered errors for the 50 first ranked triples using the score are smaller than the counterpart using only the error. Moreover, it may be observed that the 10 most frequent genes in the first 50 triples for each comparison depend on whether the score is used or not.

## Discussion

One of the goals for gene expression profiling is to identify signatures of tumor types or grades. Attempts have been made to classify gliomas based on gene expression profiling [10,15,16] using the microarray technique. Some molecular functions and biological processes are over-represented in different tumor grades. Due to important differences in the probabilistic models of microarray and SAGE techniques, the concept of a subspace credibility based on the credibility intervals of SAGE measurements was developed in order to complement the application of the strong genes technique. The microarray expression measurement is based on hybridization and optical phenomena while SAGE is based on a direct molecular counting process. Therefore, they have quite different probabilistic distributions. The strong genes technique is based on a Gaussian distribution model whereas SAGE follows a binomial distribution. In such conditions, the direct application of the strong genes technique to SAGE data is meaningful only for gene triples that present highly separated classes. The concept of credibility intervals was precisely created to evaluate the significance of the application of the strong genes technique to a binomial distribution expression measure. Furthermore, the credibility intervals are estimated from the available sample and increase for smaller sample sizes. Hence, the condition for accepting gene triples measured from small samples is very severe, i.e. they should produce very separated classes to be accepted.

The presented results revealed that, by using the score based on the spread error and the credibility, the selected triples could separate the distinct classes. The credibility analysis of strong genes was validated by comparing previous results of glioma microarray data analysis to the results of its application to glioma SAGE data. Some genes already described as related to glioma's invasion capacity by microarray studies such as SPOCK1 [17], BCL2L2 [18], EEF1A2 [15] and TMEFF1 [19] also appeared in the 50 first best triples. They are related to cell adhesion, regulation of apoptosis and translation elongation. It is important to notice that the pathways to which these genes belong could help understanding the disease progression. For example, it was shown that fibroblast growth factor-inducible 14 (Fn14) is overexpressed in migrating glioma cells in vitro and in glioblastoma multiforme clinical specimens in vivo. The biological role of Fn14 in brain cancer progression was correlated to Fn14 activation and induction of BCL2L2 mRNA and protein levels, and this effect depended on NFkappaB transcriptional activity [20]. On the other hand, some retrieved genes that were never related to gliomas progression but involved in structural/extracellular matrix-related genes or growth factor-related genes, such as PARVA and SHOC2, appear among the 10 most frequent genes in the 50 best triples only when the score with credibility is applied. Besides, because of the fact that structural/extracellular matrix-related genes or growth factor-related genes have an important role in glial tumors [21], it was recently suggested that SHOC2 function is essential for activation of MAPK pathway by growth factors [22].

**Figure 4:**
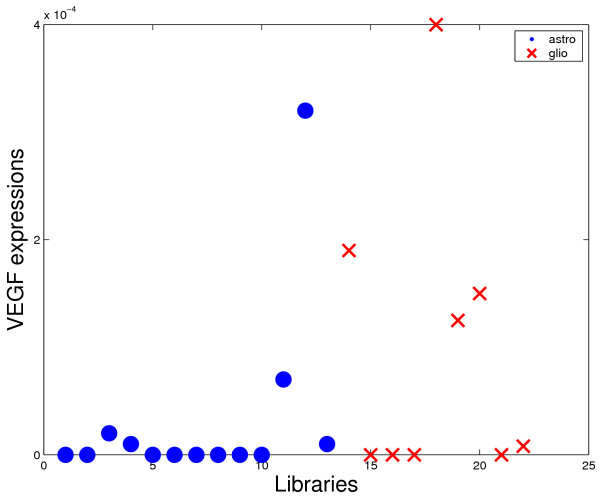
**Discrimination power comparison**. Expression of VEGF for each library of the comparison astrocytomas II and III versus glioblastoma.

**Figure 5:**
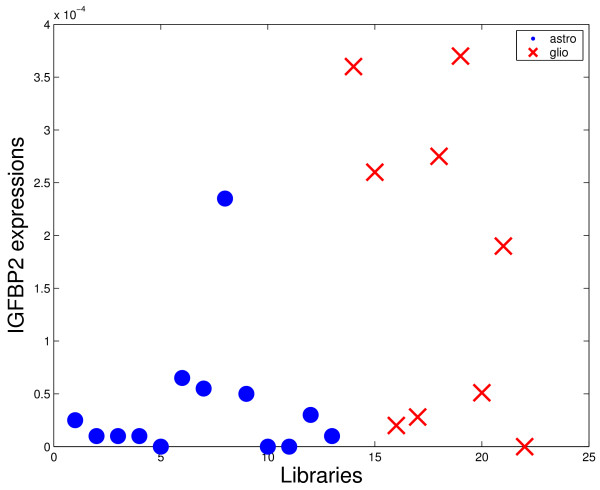
**Discrimination power comparison**. Expression of IGFBP2 for each library of the comparison astrocytomas II and III versus glioblastoma.

**Figure 6:**
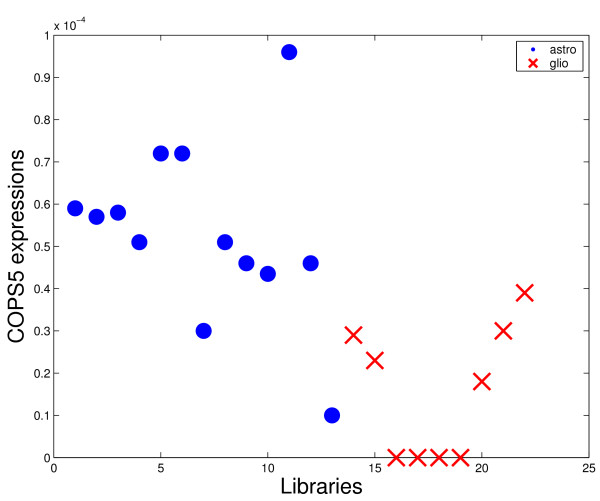
**Discrimination power comparison**. Expression of COPS5 for each library of the comparison astrocytomas II and III versus glioblastoma.

**Figure 7:**
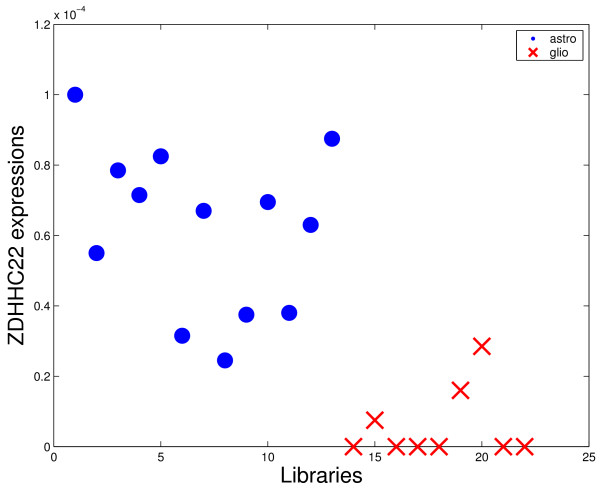
**Discrimination power comparison**. Expression of ZDHHC22 for each library of the comparison astrocytomas II and III versus glioblastoma.

However, other genes usually related to gliomas such as VEGF or IGFBP2 were not present in the first triples. There are some reasons for this fact. The first is that the VEGF was excluded from the analysis by the adopted criterion of tags exclusion (see Methods section). Even so, this gene presented low representativity (large number of zeros for both considered classes in all comparisons). For the case of IGFBP2, this gene was included in the set of analyzed genes, but the discrimination power is not enough to appear in the best triples. Figures 4-7 shows four graphics for the *astrocytoma *II and III versus *glioblastoma *comparison that illustrate the difference of the discrimination power of VEGF and IGFBP2 with respect to COPS5 and ZDHHC22. The two last ones presented the highest frequencies in the first 50 best triples for the considered comparison.

The difference of the results presented by microarray and SAGE methods may be explained by the particularities of each method. The Spearman correlation between these two methods is *r *= 0.6 in both absolute expression and comparative analysis [23]. The microarray and SAGE results tend to be consistent when both samples are large enough and there are triples that produce highly separated classes. In other conditions, such results may not be consistent. In the case of the results here reported, the small SAGE sample size explains why some well known glioma genes were out of our best triples.

## Conclusion

Even using the credibility intervals, which is feasible for SAGE but not for microarray data, it is difficult to define the "best triple" because of the small sample size and high data variability. Because of such statistical limitations, it is more realistic to identify larger sets of genes in the top position triples for posterior analysis. The introduced methodology allows ranking a given number of best candidates to be subsequently analyzed in a complementary way. The introduced method is suitable to define the triples that perform a good separation of the classes using SAGE since it is possible to use data from different laboratories. It is important to note that our model may also be applied for other counting methods such as Massive Parallel Signature Sequencing (MPSS) or the recent Sequencing-By-Synthesis (SBS) technique. Moreover, some of the best triples identified by the proposed method may be useful in the future to generate classifiers.

## Methods

### SAGE data

The input tables consist of 24 SAGE libraries, each corresponding to one sample (2 from normal brain, 4 from *astrocytoma *grade II, 9 from *astrocytoma *grade II and 9 from *glioblastoma*). These libraries were obtained from SAGE Genie [24,25]. Only full length genes which have a unique 3' tag (not present as a 3' tag or an internal tag of another full length) were considered. Tags that have 8 or more A in their sequence were eliminated because ambiguous tags have the potential to represent the sum of expression of several genes, thus artificially increasing the observed tag frequency. The tag AAAAAAAAAA, which can be derived from the poly(A) tail of many transcripts, is seen at a relatively high frequency in most SAGE libraries, which explains why we decided to avoid such tags. 28370 full lengths were initially considered. The application of the above filtering criterion leads to 17599 genes that have been analyzed. The expression abundance *e*_*ij *_of a tag *i *in a library *j *is the number of counts of the tag *i *in the corresponding library *j *divided by the number of counts of all tags in the library *j*.

### Strong genes technique

Due to the small number of samples of the SAGE experiments focused here, we could not afford to leave out a subset of the data for testing. We decided then to use bolstered error with linear discriminant analysis (LDA) and normal kernels to evaluate the quality of gene subsets, as suggested by Kim *et al*. [10] and further developed by Braga-Neto and Dougherty [26].

One of the main problems while selecting good genes and designing classifiers is that the usual error rate estimates, like resubstitution, leave-one-out or ten fold cross validation, present high variance [27]. This is specially relevant in a small sample setting where it is not possible to leave data out from classifier design for testing [28]. In order to alleviate such problems, Kim *et al*. [10,29] proposed to spread each sample using a kernel distribution. In a follow up paper, Braga-Neto and Dougherty have generalized this technique and shown how to best choose the distribution parameters [26]. The general technique was called *bolstered error estimation*. Genes that give rise to a classifier with small bolstered error are called *strong genes*. The idea of bolstered error estimation is to spread each sample using a fixed probability distribution. We have used a circular normal distribution with fixed variance *σ*^2 ^as in [10]. The bolstered error of a classifier is the mass of the probability distribution that is misclassified. Naturally if we increase *σ*^2^, the estimated error will increase. Since we use a circular normal distribution, the optimal classifier and the respective bolstered error can be computed analytically for a fixed *σ*^2 ^and a gene subset [10]. We denote this bolstered error as *ε*_*σ*_.

For *σ *= 0, *ε*_0 _is equal to the resubstitution error of the computed linear classifier, a low-biased error estimator. Model-based simulation investigated in [10] suggests that for *σ *= 0.4, *ε*_*σ *_is an unbiased error estimator and the bias increases with *σ*. We focused on *σ *= 0.4 in order to use conservative error estimates as in [10]. Another important property of the bolstered error estimation is that its variance decreases with increasing *σ*. This fact ensures that the estimator variance is smaller than the variance for resubstitution and leave-one-out. Leave-one-out variance is high for small sample sets and it is usually larger than resubstitution variance, which is equal to the bolstered error for *σ *= 0. Moreover the small number of samples in each comparison forced us to concentrate on very small gene sets: three in our case, i.e. gene triples were searched. Finally, in order to use the algorithm explained in [10], an important practical issue had to be addressed, i.e. computational time. The very high number of tags or genes (17599 in our experiments) translates into a huge number of possible triples: 1.49 × 10^12^. Even with the analytic solution for the classifier and bolstered error estimate, the time required to explore all triples for a fixed *σ *would be close to a year on a typical desktop computer. We decided then to use the pre-processing algorithm proposed by Silva [11] based on linear support vector machines. This technique looks for a small group of genes that are able to separate the data with high quality, in the sense that the samples of different classes are linearly separable and far away from each other. However, there is no way to control the groups size and they turn out to be too large, usually a few tens. After selecting a hundred genes using the pre-processing algorithm, a full search of the possible triples is carried out using the bolstered error. It was shown in [11] that the pre-processing usually keeps the best gene subsets even though the computation time takes only some minutes. The criterion used by SVM to choose good genes, a geometric distance of the two classes to the separating hyperplane, is intuitively related to the bolstered error estimator.

Actually, if the genes of a class are close to the decision surface, the mass of a distribution centered on such samples that is incorrectly classified is expected to be high. On the other hand, if the distance is large, the incorrectly classified portion of such distributions should decrease. This correlation has been empirically verified in many tests described in [11]. It is worth noting that the SVM does not process each gene isolated. It searches the space of all genes at the same time, implicitly taking all possible combinations into account. The SVM finds a small group that presents the best discriminatory power according to the ∞-norm criterion.

Considering that SAGE data usually contains dozens of samples with thousands of tag counts, the strong genes technique adopted here looks only for those triples that provide the best linear separation of the classes. It is easy to see that in this case there are many possible linear classifiers, unless the sample is very peculiarly disposed in space. Finally, more expressive classifiers tend to overfit the sample more easily and should be avoided. In cases where the number of samples is large enough to justify the use of nonlinear classifiers, nonlinear Support Vector Machines based on (nonlinear) kernel functions may be adopted to generalize our approach.

### Credibility intervals

Once SAGE expression data is obtained by sampling (i.e. counts of observed tags), it is important to have a credibility measure for each expression based on the total number of tags counted in each library. This process may be modeled by a Beta probability density function (pdf) [13]. Let *x *be the number of counts of a given tag and *b *be the total number of counts for all tags in a given library. The credibility interval around *x *is calculated using the Beta pdf:



where *f*(*i*) indicates the probability of the real number of counts be *i *given *b *and *x*.

Once a credibility value C
 MathType@MTEF@5@5@+=feaafiart1ev1aaatCvAUfKttLearuWrP9MDH5MBPbIqV92AaeXatLxBI9gBamrtHrhAL1wy0L2yHvtyaeHbnfgDOvwBHrxAJfwnaebbnrfifHhDYfgasaacH8akY=wiFfYdH8Gipec8Eeeu0xXdbba9frFj0=OqFfea0dXdd9vqai=hGuQ8kuc9pgc9s8qqaq=dirpe0xb9q8qiLsFr0=vr0=vr0dc8meaabaqaciaacaGaaeqabaWaaeGaeaaakeaaimaacqWFce=qaaa@3825@ is fixed, 0 <C
 MathType@MTEF@5@5@+=feaafiart1ev1aaatCvAUfKttLearuWrP9MDH5MBPbIqV92AaeXatLxBI9gBamrtHrhAL1wy0L2yHvtyaeHbnfgDOvwBHrxAJfwnaebbnrfifHhDYfgasaacH8akY=wiFfYdH8Gipec8Eeeu0xXdbba9frFj0=OqFfea0dXdd9vqai=hGuQ8kuc9pgc9s8qqaq=dirpe0xb9q8qiLsFr0=vr0=vr0dc8meaabaqaciaacaGaaeqabaWaaeGaeaaakeaaimaacqWFce=qaaa@3825@ < 1, the credibility interval extreme values *t*_1 _and *t*_2 _are obtained by integrating *f*(*i*) around its mode so that *f*(*t*_1_) = *f*(*t*_2_) and

∫t1t2f(i)di=C.
 MathType@MTEF@5@5@+=feaafiart1ev1aaatCvAUfKttLearuWrP9MDH5MBPbIqV92AaeXatLxBI9gBamrtHrhAL1wy0L2yHvtyaeHbnfgDOvwBHrxAJfwnaebbnrfifHhDYfgasaacH8akY=wiFfYdH8Gipec8Eeeu0xXdbba9frFj0=OqFfea0dXdd9vqai=hGuQ8kuc9pgc9s8qqaq=dirpe0xb9q8qiLsFr0=vr0=vr0dc8meaabaqaciaacaGaaeqabaWaaeGaeaaakeaadaWdXaqaaiabdAgaMjabcIcaOiabdMgaPjabcMcaPiabdsgaKjabdMgaPjabg2da9GWaaiab=jq8djabc6caUaWcbaGaemiDaq3aaSbaaWqaaiabigdaXaqabaaaleaacqWG0baDdaWgaaadbaGaeGOmaidabeaaa0Gaey4kIipaaaa@486F@

The distribution's mode coincides with the number of counts of a given tag (i.e. it occurs at *i *= *x*) whenever a non-informative uniform *a priori *distribution is assumed.

A credibility index is defined to characterize the discrimination power of each gene triple for libraries related to two different biological states. The credibility index for a given gene triple (selected by the strong gene method) is calculated as follows. Firstly, the credibility interval for a given credibility value C
 MathType@MTEF@5@5@+=feaafiart1ev1aaatCvAUfKttLearuWrP9MDH5MBPbIqV92AaeXatLxBI9gBamrtHrhAL1wy0L2yHvtyaeHbnfgDOvwBHrxAJfwnaebbnrfifHhDYfgasaacH8akY=wiFfYdH8Gipec8Eeeu0xXdbba9frFj0=OqFfea0dXdd9vqai=hGuQ8kuc9pgc9s8qqaq=dirpe0xb9q8qiLsFr0=vr0=vr0dc8meaabaqaciaacaGaaeqabaWaaeGaeaaakeaaimaacqWFce=qaaa@3825@ is calculated for each gene of each library. For each library, the respective three credibility intervals define the vertices of a box. If there is no box intersections between the libraries of one biological state with the library boxes of the other biological state, then the credibility index is increased. In case of non-empty intersection, the credibility index is decreased. A binary-search like procedure is applied to calculate the credibility index from the libraries data. This binary-search procedure starts with C
 MathType@MTEF@5@5@+=feaafiart1ev1aaatCvAUfKttLearuWrP9MDH5MBPbIqV92AaeXatLxBI9gBamrtHrhAL1wy0L2yHvtyaeHbnfgDOvwBHrxAJfwnaebbnrfifHhDYfgasaacH8akY=wiFfYdH8Gipec8Eeeu0xXdbba9frFj0=OqFfea0dXdd9vqai=hGuQ8kuc9pgc9s8qqaq=dirpe0xb9q8qiLsFr0=vr0=vr0dc8meaabaqaciaacaGaaeqabaWaaeGaeaaakeaaimaacqWFce=qaaa@3825@ = 0.5 and is repeated *m *times to produce the final credibility index. Larger *m *values lead to more accurate credibility values. In our experiments, *m *= 7 was adopted.

### Signature genes identification

A pipeline system has been implemented in order to integrate the aforementioned SAGE analysis procedures. The system takes as input the selected data as described in the previous section (SAGE data) and pre-processes the data to build a matrix with these libraries associated to the selected comparison made by the user (e.g. *astrocytoma *III versus *glioblastoma*). Then, the strong genes selection procedure is applied to this matrix, producing a table with 1000 best gene triples ordered by the bolstered error. Although 1000 triples represent a much smaller set with respect to all possibilities, it is too long to be analyzed by human inspection. In order to identify triples that are potential candidates for differential diagnosis of these tumor types, the score given by Equation 3 is calculated and assigned to each triple.

Scorei=(Ei−min(E)NE)2+(2Ci−max(C)NC)2,
 MathType@MTEF@5@5@+=feaafiart1ev1aaatCvAUfKttLearuWrP9MDH5MBPbIqV92AaeXatLxBI9gBaebbnrfifHhDYfgasaacH8akY=wiFfYdH8Gipec8Eeeu0xXdbba9frFj0=OqFfea0dXdd9vqai=hGuQ8kuc9pgc9s8qqaq=dirpe0xb9q8qiLsFr0=vr0=vr0dc8meaabaqaciaacaGaaeqabaqabeGadaaakeaacqWGtbWucqWGJbWycqWGVbWBcqWGYbGCcqWGLbqzdaWgaaWcbaGaemyAaKgabeaakiabg2da9maakaaabaWaaeWaaeaadaWcaaqaaiabdweafnaaBaaaleaacqWGPbqAaeqaaOGaeyOeI0ccbiGae8xBa0Mae8xAaKMae8NBa4MaeiikaGccbeGae4xrauKaeiykaKcabaGaemOta40aaSbaaSqaaiabdweafbqabaaaaaGccaGLOaGaayzkaaWaaWbaaSqabeaacqaIYaGmaaGccqGHRaWkdaqadaqaaiabikdaYmaalaaabaGaem4qam0aaSbaaSqaaiabdMgaPbqabaGccqGHsislcqWFTbqBcqWFHbqycqWF4baEcqGGOaakcqGFdbWqcqGGPaqkaeaacqWGobGtdaWgaaWcbaGaem4qameabeaaaaaakiaawIcacaGLPaaadaahaaWcbeqaaiabikdaYaaaaeqaaOGaeiilaWcaaa@57E9@

where *E*_*i *_is the bolstered error of triple *i*, **E **is the vector of these errors for all 1000 triples, *C*_*i *_is the credibility value of triple *i*, **C **is the vector of these credibilities for all 1000 triples, *N*_*E *_= *max*(**E**) - *min*(**E**) and *N*_*C *_= *max*(**C**) - *min*(**C**). Lower scores lead to better triples. All the triples are ranked by this score and we have chosen the first 50 from each comparison for posterior analysis (see Results and Discussion sections).

The system produces a series of output information in HTML form: a table with all selected gene triples, the number of libraries, the error, the distance between the classes (i.e. biological states), the number of occurrences of each gene in the list, the credibility value and the score defined by Equation 3 (see an example in Figure 1). URL's for 3D plots of each triple are produced by the system (Figure 2).

Three-dimensional plots of the credibility interval boxes are also generated (Figure 3). All output features were designed to help the biological interpretation by the biomedical experts.

## Authors' contributions

JB, RMCJ, DCMJ, PJSS, CHJ and HB analyzed the initial problem and conceived the general framework of the proposed approach. DCMJ worked on most implementation details to integrate the whole pipeline. JB, RMCJ and DCMJ worked on the development and implementation issues of the credibility intervals method. PJSS and CHJ developed and implemented the strong genes and SVM method. HB and DFCP proposed the final score used to rank the genes and made the biological interpretation of the results. All authors participated in the production of the manuscript.
